# Examining the physico-chemical, structural and thermo-mechanical properties of naturally occurring Acacia pennata fibres treated with KMnO_4_

**DOI:** 10.1038/s41598-023-46989-x

**Published:** 2023-11-24

**Authors:** K. R. Jaya Sheeba, Retnam Krishna Priya, Krishna Prakash Arunachalam, S. Shobana, Siva Avudaiappan, Erick Saavedra Flores

**Affiliations:** 1 PG & Research Department of Physics, Holy Cross College (Autonomous), Nagercoil, Affiliated to Manonmanium Sundaranar University, Tirunelveli, Tamil Nadu 629004 India; 2 Department of Civil Engineering, University College of Engineering Nagercoil, Anna University, Nagercoil, 629004 India; 3 Green Technology and Sustainable Development in Construction Research Group, Van Lang School of Engineering and Technology, Van Lang University, Ho Chi Minh City, Viet Nam; 4 Departamento de Ingeniería Civil, Universidad de Concepción, 4070386 Concepción, Chile; 5 Centro Nacional de Excelencia Para la Industria de la Madera (CENAMAD), Pontificia Universidad Católica de Chile, Av. Vicuña Mackenna 4860, 8331150 Santiago, Chile; 6 Department of Physiology, Saveetha Dental College and Hospitals, SIMATS, Chennai, 600077 India; 7 Departamento de Ingeniería en Obras Civiles, Universidad de Santiago de Chile, Av. Ecuador 3659, Estación Central, Santiago, Chile

**Keywords:** Civil engineering, Plant ecology

## Abstract

Natural fiber is a viable and possible option when looking for a material with high specific strength and high specific modulus that is lightweight, affordable, biodegradable, recyclable, and eco-friendly to reinforce polymer composites. There are many methods in which natural fibres can be incorporated into composite materials. The purpose of this research was to evaluate the physico-chemical, structural, thermal, and mechanical properties of Acacia pennata fibres (APFs). Scanning electron microscopy was used to determine the AP fibers' diameter and surface shape. The crystallinity index (64.47%) was discovered by XRD. The irregular arrangement and rough surface are seen in SEM photos. The findings demonstrated that fiber has high levels of cellulose (55.4%), hemicellulose (13.3%), and low levels of lignin (17.75%), which were determined through chemical analysis and validated by Fourier Transform Infrared Spectroscopy (FTIR). By using FTIR, the functional groups of the isolated AP fibers were examined, and TG analysis was used to look into the thermal degrading behaviour of the fibers treated with potassium permanganate (KMnO_4_) Due to their low density (520 kg/m^3^) and high cellulose content (55.4%), they have excellent bonding qualities. Additionally, tensile tests were used for mechanical characterisation to assess their tensile strength (685 MPa) and elongation.

## Introduction

Our living planet Earth is the source of abundant wealth and resources. It provides shelter for over seven million species of plants and animals. Today's diverse cellulose fibers, which have evolved over the past few decades and include flax, hemp, sisal, cotton, kenaf, jute, bamboo, coconut, and date palm, provide a variety of advantages over synthetic fibers (mostly glass, carbon, and plastic) due to their renewable nature^1, 2^. A number of natural fiber materials can be distinguished by their place of origin in nature. According to specific classifications made by researchers^3–8^ these materials fall into three categories: fibers produced from animals, minerals, and plants. These fibers are used in a variety of composite material manufacturing processes^9–13^. In comparison to standard reinforcing materials, natural fibres have greater thermal and acoustic insulating qualities, an acceptable specific strength, cheap cost, and low density^14–18^. The maturity and origin of the plant, as well as the methods and techniques used to extract the fiber from the stem, still affect the mechanical properties of the fibers^19^. For every good alternative material without sacrificing the mechanical properties of the fiber, these are some better fiber-yielding plants that are reasonably priced. Acacia pennata (AC) is one such plant, and it is most readily available in tropical areas of India. In contrast, natural fibers were safe, renewable, biodegradable, eco-friendly, and affordable with high specific strength^20, 21^. Ripples of applications are found in natural fibers from household little equipment’s to aviation. It is because of the efficient properties possessed by these natural composites like light weight, high aspect ratio, low density, soundproof, good thermal and mechanical properties, biodegradability etc.

According to the literature review, natural fibers will become more significant in the future since they are readily available, recyclable, and environmentally friendly^22^. For automotive applications, polymer composites with various fillers and / or reinforcements are often employed. In order to ensure that the overall cost of the vehicle is lower and that the automobile manufacturing process is more sustainable, numerous such composites have recently been created for use in interior and exterior sections of cars^23, 24^. The usefulness of a fiber for commercial purposes is decided by features such as length, strength, pliability, elasticity, abrasion resistance, flexibility, etc., aside from economic considerations. Younger fibers from plants tend to be stronger and more elastic than the older ones^25, 26^. The cellulose is the main content of fibers which corresponds to the crystalline nature of the fibers and the presence of hemicellulose, pectin, lignin should be wiped or reduced by processing for improvements. Also, the antibacterial property is a noted one. Animal, mineral, and plant fibers are specifically the three groups into which natural fibers fall^27^. The utilisation of natural fibres for the purpose of filling and reinforcing thermoplastic polymers is currently the most prevalent method of reinforcement in today's world. Tensile strength is the most crucial mechanical characteristic of natural fibers that makes them ideal for creating composite materials. Most naturally occurring fibers fall short in the hydrophilic department, which results in poor chemical resistance, subpar mechanical qualities, and porous structures that restrict the engineering uses of these materials^28, 29^. When choosing a specific fibre to be utilised in a composite material or in any other industrial application, the density, young's modulus, elongation, and stiffness are other crucial parameters that must be taken into account^30–32^. Natural fibres make excellent insulation against heat, sound, and electricity. They are also easily burnable and biodegradable^33^. For instance, composites have been utilised in bumpers, roofs, doors, panels, seats of cars, buses, and other vehicles in the automotive sector. Researchers are now interested in improving mechanical qualities like compression, tensile, flexural, or impact strength, as well as wear behaviour, which represent the great achievement of good materials^34^. Particularly, composite materials are being created and modified in an effort to enhance existing products as well as to offer new ones in a sustainable and ethical manner^35^.

The goal of the current study is to examine and compare the physical, chemical, thermal, mechanical, and morphological analysis of Acacia pennata fibers (APFs) with that of other probable natural fibres mentioned in the literature. In order to determine the APFs bonding properties, the surface roughness of the material was also measured using a three-dimensional non-contact surface roughness tester. To determine the contents of cellulose, hemicellulose, lignin, pectin, wax, moisture, and ash, a chemical analysis was carried out. In addition, thermogravimetric analysis, X-ray diffraction (XRD), and Fourier transform infrared (FTIR) examinations were used to analyse the crystalline phases and compounds.

## Materials and methods

### Materials

The Acacia pennata fiber was treated using destilled water, NaOH pellets, and a potassium permanganate pellets in acetone. This was acquired from Premier Chemicals in Nagercoil, Kanniyakumari district, Tamil Nadu, India.

### Extraction method of Acacia pennata fiber from the plant

**Figure 1:**
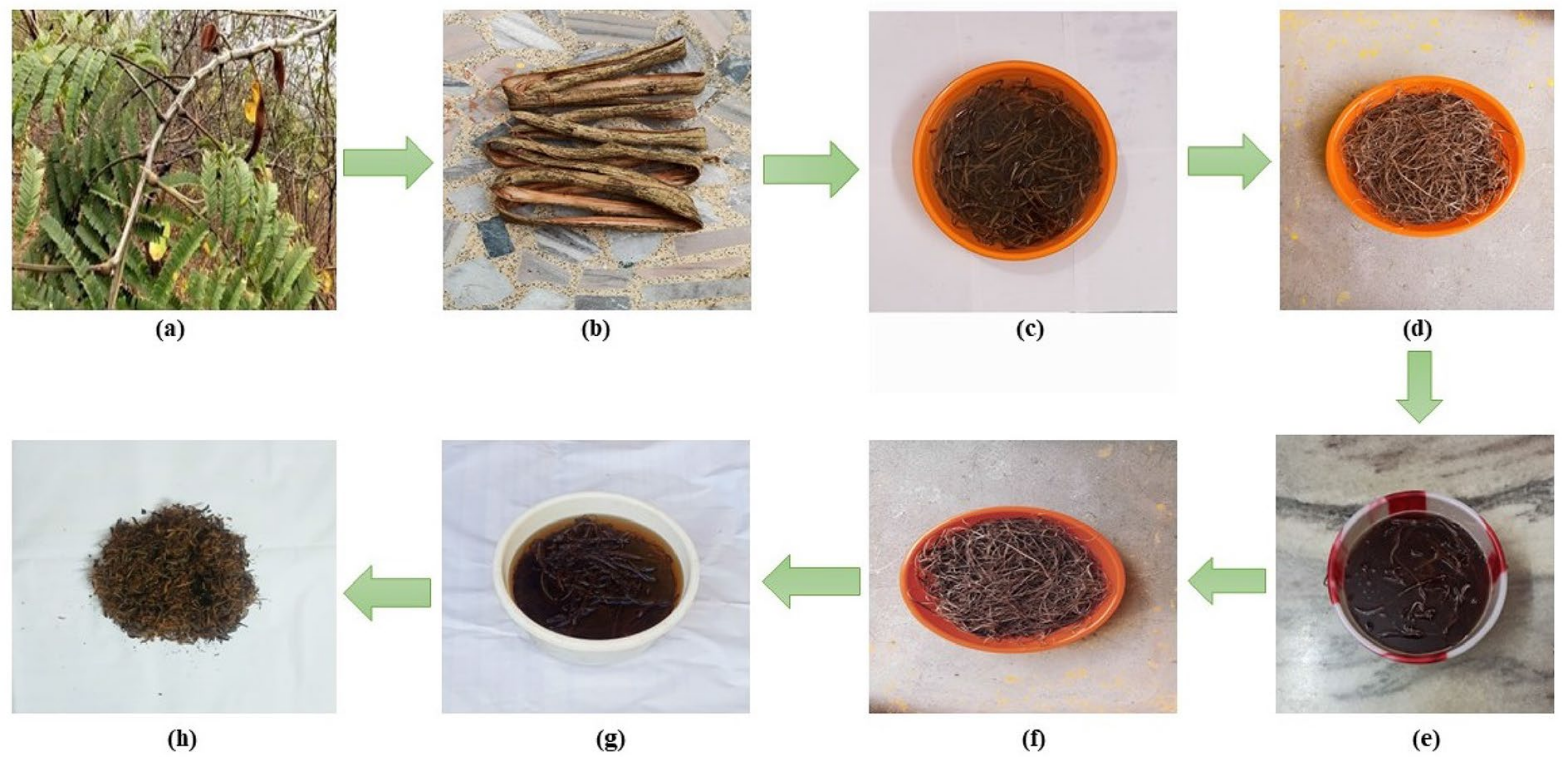
Images of an (**a**) AP plant, (**b**) AP stem fiber, (**c**) water retting, (**d**) untreated dried AP fibers, (**e**) AP fibres treated with NaOH. (**f**) Dried NaOH treated AP fibers, (**g**) AP fibres treated with KMnO4, (h) Dried KMnO4 treated AP fibers.

Acacia pennata is a large scrambling or climbing shrub. It can grow up to a height of almost 100 m. Its trunk and branches are prickly and smooth. The stem of Acacia pennata fibers were gathered from Tamilnadu-Kerala border (near Panachamoodu and Vellarada area). The fiber has been separated from its bark of the plant and then allowed to dry at ambient temperature (27 °C) for few days. The dried fibers are treated with distilled water for about 20 min for microbial degradation before treating the fiber with alkali (NaOH). The dried fiber sections were pre-treated with NaOH aqueous solution and the potassium permanganate (KMnO_4_) solution was prepared with the help of acetone and KMnO_4_ pellets. The importance of this treatment for improving surface properties was also addressed in depth. This allowed us to reduce the hydrophilic capacity and increase the adhesion between the fibers and polymers^36^. The fibers were soaked in this (0.1 M NaOH) solution for 20 min. The fibers were removed after 20 min and dried for 10 to 15 days at room temperature (27 °C). After drying, the fiber sections are immersed in KMnO_4_ solution for about 15 minitues^37^. Then, these fibers were allowed to dry at ambient temperature. The dried AP fibers were chopped into powder form or broken into little fiber strips based on the need of analysis. KMnO_4_ treated AP fibers were packed in zip-lock cover and store in room temperature. Figure 1 shows the pictures of Acacia pennata plant fiber and its chemical treatments.

### Experimental techniques

#### Powder XRD technique

Powder X-ray diffraction (PXRD) is a method for determining if a material is crystalline or amorphous^38^. It is a quick analytical method that can reveal the size of unit cells and is mostly used to determine a crystalline material's phase. Copper (Cu) is the most common target material for powder x ray diffraction, and the powder sample were subjected to X-ray diffraction using CuKα (i.e., CuKα is the emission of copper) radiation with a wavelength of 1.5406 nm in a Bruker x-ray diffractometer. A 2θ range X-ray diffraction examination was performed with angles ranging from 3° to 70°. In the spectrum of the APFs, the integrated intensities of the Bragg peaks were recognised, and their crystallinity indices were calculated. The crystallinity index (CI) of the natural fiber was measured using the traditional peak height technique developed by Segal et al^39^.

1 $${\text{CI}} = \left[ {\left( {{\text{H}}_{002} - {\text{ H}}_{{{\text{am}}}} } \right)/{\text{H}}_{002} } \right] \times 100\%$$

where, H_002_ is the height of the crystalline peak situated around 22° and 23°; H_am_ is the height of amorphous peak situated around 14° and 16°.

The crystallite size (CS) of the natural fiber were calculated through the following equation^40^;

2 $${\text{CS}} = [0.89\lambda /\beta \cos \theta ]$$

where, β is the full-width at half-maximum (FWHM) value of peaks; θ is the Bragg’s angle^41^.

#### FTIR techniques

A crucial method for locating significant groups is Fourier-transform infrared (FTIR) spectroscopy^42^. “Perkin Elmer Spectrum Two” FTIR spectrometer with a scan rate of 32 per minute, a resolution of 2 cm^−1^, and a wave number range between 4000 cm^−1^ and 400 cm^−1^ were used to obtain the FTIR spectra of the APFs.

#### SEM techniques

A scanning electron microscope (SEM) with EDS is used to investigate the topography and morphology of the surfaces of materials as well as biological samples. EDS is used to facilitate elemental recognition. A concentrated electron beam is used to scan a sample's surface in a scanning electron microscope (SEM), which creates images of the sample. The spatial correlations between the various matrices' and reinforcement fibers' constituent parts have been clarified by SEM studies^43^. Using a scanning electron microscope, the surface morphology of APFs was studied (JEOL JSM-6390LV)^44–46^. Magnification of the JEOL JSM-6390LV is on the order of 3,00,000 × with high resolution 3.0-nm, where fine details of the specimens can be observed.

#### Thermo-gravimetric techniques

Thermo-gravimetric analysis by Perkin Elmer was used to access the thermal stability behaviour of APFs. The curve plots the temperature difference between the reference material and the sample material against time or temperature. The amount and rate of change in a material's weight as a function of temperature or time in an environment of nitrogen, helium, air, another gas, or in a vacuum are measured using thermo-gravimetric analysis (TG). The method can identify materials that experience weight gains or loss as a result of oxidation, dehydration, or other processes^47^.

#### Density using pycnometer

The thickness (density) of natural fibers is frequently measured with a pycnometer^48^. The fiber sample is simply dried at room temperature before use to remove moisture^49^. If moisture remains in the fiber material, a vacuum desiccator can be employed to eliminate it completely. The samples are then thoroughly pulverised and placed in the pycnometer to measure the density^50^. Toluene is used as an immersion solvent when measuring the densities of untreated and KMnO_4_ treated AP fibres in accordance with ASTM D578-89 standard. The fibres must be soaked in toluene for at least two hours before being weighed to assess their density. Density of APF is derived from;

3 $$\rho f=\frac{({\text{m}}_{2}-{\text{m}}_{1})}{[\left({\text{m}}_{3}-{\text{m}}_{1}\right)-\left({\text{m}}_{4}-{\text{m}}_{2}\right)]}*\rho t$$

where, m_1_—mass of dry empty pycnometer (g); m_2_—mass of pycnometer + fiber (g); m_3_—mass of pycnometer + toluene (g); m_4_—mass of pycnometer + toluene + fiber (g); ρt—density of toluene (0.867 g/cm^3^); ρf—density of natural fiber in g/cm^3^^51–57^.

#### CHNS (elemental) analysis techniques

Using a CHNS analyzer, model Elementar Vario EL III Instruments based on the principle of Dumas's method^58^, which involves the complete and quick oxidation of the sample by "flash combustion," one may determine the percentages of C, H, N, S, (carbon, hydrogen, nitrogen, sulphur and oxygen elements) in organic compounds. To provide carbonate and organic carbon and to get a general idea of the composition of the organic matter (i.e., to distinguish between marine and terrigenous sources, based on total organic carbon / total nitrogen [C/N] ratios), elemental analyses of total nitrogen and carbon (and sulphur) are conducted.

#### Tensile strength analysis

There are various reasons to undertake tensile tests. Typically, Zwick/Roell^59^ specimens are used for the tensile test^60^. The maximum stress that the material can withstand or the stress required to generate substantial plastic deformation are two ways to assess the strength of an object of interest. A computerised tensile testing machine was used for the tensile testing analysis. The tensile mechanical characteristics of a material are described in depth via tensile testing and also these tests were performed with ASTM-D412 international standards^59^. These characteristics can be represented graphically as a stress/strain curve to display information such as the point at which the material failed and to provide information on characteristics such the elastic modulus, strain, and yield strength^61^. The tests are conducted at a temperature of 21 °C, a cross-head speed of 30 mm/min, and a relative humidity of 65 ± 2%^62^. To confirm the accuracy of the tensile test, 10 fibers, each 50 mm long, are tested^63–71^. Records are kept of the average tensile strength, elongation at break, and strain rate. The following empirical relationship governs the tensile strength of the AP fibers;

4 $$T=\frac{F}{A}$$

where; F is the force in Newtons, A is cross-sectional area in mm^2^ and T is the tensile strength in MPa^72, 73^.

Stress–strain curves are used to evaluate the mechanical characteristics of AP fibers, including their tensile strength and percentage of elongation^70, 74^. A microscope is used to conduct a comprehensive longitudinal direction of the chosen acacia pennata fibers in order to measure their average diameter. Additionally, from the SEM images, the thickness of the fibers is calculated using the ImageJ software^59^. Nearly the same diameter was obtained by both techniques. The shape of APFs cross section is round^53, 75–77^. The angle formed by the helical winding orientations of cellulose microfibrils is known as the microfibril angle (MFA). A plant fibers strength and stiffness are often affected by the amount of cellulose and the spiral-shaped wink. The Global deformation equation is used to determine the microfibrillar angle (α) of AP fibers.

5 $$\epsilon =\mathrm{ln}\left(1+\frac{\Delta L}{\text{Lo}} \right)=-\mathrm{ln}(cos\alpha )$$

where ϵ—strain, α—micro-fibril angle (degree) and (∆L/Lo)—ratio of elongation^50, 52–54, 78, 79^.

The term “Youngs modulus” is used to describe a material's resistance to elastic deformation under load. It displays the strength of a material, to put it another way.

6 $$E=\frac{\sigma (Tensile strength)}{\epsilon (strain)}=\frac{(\frac{F}{A})}{(\Delta L/L{\text{o}})}$$

where, F—applied force; E—young’s modulus (GPa) of the fiber; A—cross-sectional area of the fiber and ∆L/Lo—ratio of elongation^74^.

The elongation at break is the ratio of the modified length to the original length when a test specimen is fractured. It demonstrates how naturally occurring plant fiber may withstand changes without breaking. It is possible to calculate the elongation at break of the AP fiber in accordance with ISO/IEC 17,025^80, 81^ tensile test.

#### Chemical analysis test

Chemical analysis tests were used to identify the chemical composition of natural fibers and how it affected their mechanical qualities. To study the percentages of chemical compositions (cellulose, hemicelluloses, lignin, pectin, wax, and ash) which present in the natural fiber using chemical analysis. These are the primary components of natural fibers. All chemical analysis tests were performed in accordance with ASTM-D3822 and IS199 international standards^82^. Both natural and synthetic fibers are currently used in the production of engineering materials. As a result, their mechanical and thermal characteristics depend on the environment. Therefore, in-depth research must be done to analyse the characteristics of natural fibers.

### Specimen collection

The Acacia pennata plant has spread and grown throughout Kerala and Tamil Nadu in India. Acacia pennata plant for the research purposes are collected from the authors farm at Panchamoodu and Vellarada.

### Guidelines and regulations

All testing was performed in accordance with the relevant ISO standards. The methods and procedures used were compliant with the guidelines and regulations outlined in the ISO standard to ensure accurate and reliable results.

## Results and discussion

### Powder XRD analysis

**Figure 2:**
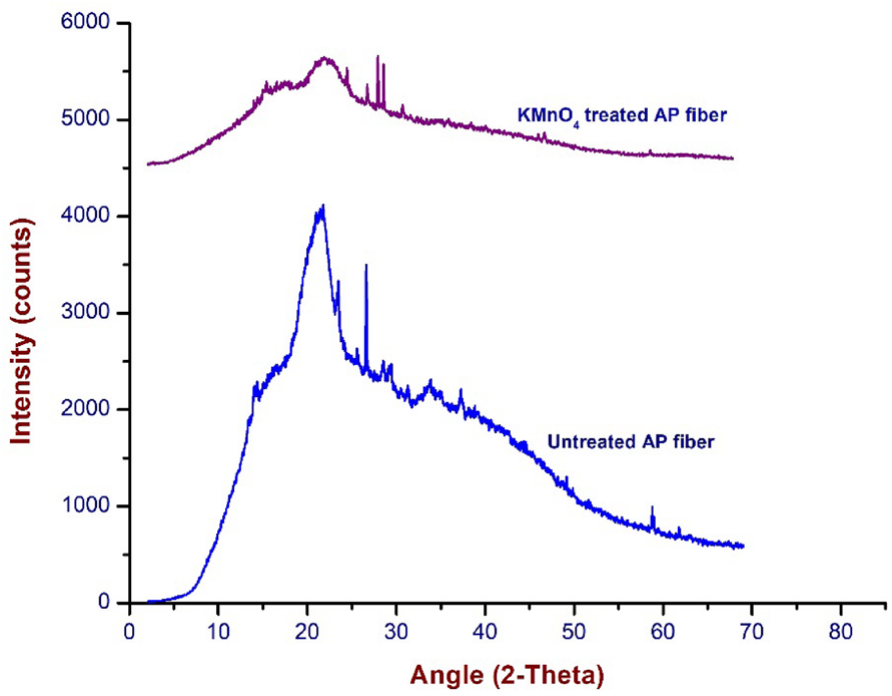
PXRD patterns of untreated and KMnO_4_ treated AP fiber.

Figure 2 depicts the X-ray diffraction (XRD) pattern of the untreated and KMnO_4_-treated AP fibers. It (untreated APF) shows the crystalline peak (22.59°) on the crystallographic plane (002) and amorphous peak (15.25°) on the lattice plane (110), which demonstrates the semi-crystalline nature^77^. This is because hemicellulose, lignin, and pectin are present. Two well-defined diffraction peaks are observed in KMnO_4_ treated APFs around 2θ = 22.73° and the amorphous peak at 2θ = 15.37° include lignin, pectin, hemicellulose, and amorphous cellulose, which contains a larger percentage of amorphous fraction^83^. The crystallinity index of KMnO_4_ treated fiber was determined as 64.47%. The high crystallinity index of AP fiber was caused by the efficient removal of contaminants and hemicellulose. It was slightly increased when compared to untreated (46.52%) AP fiber. The CI of permanganate treated AP fibers are significantly greater than Kapok (45%) fiber and substantially lower than crushed guadua (65.3) fiber, jute (71%) and hemp (88%) fiber^31, 84^. However, using the well-known scherrer formula, the crystallite size (CS) of the KMnO_4_ treated APFs was found to be 6.75 nm and it was higher than that of untreated (1.9 nm) AP fiber. The crystalline size value is lower than that of ramie fibres (16 nm) and higher than that of cotton fibers (5.5 nm), tamarindus indica fruit fibers (5.73 nm), ferula communis (1.6 nm), and carbon fibers (0.669 nm)^85, 86^. Table 1 represents the comparison of CI and CS values of raw and KMnO_4_ treated APFs with other natural fibers.

**Table 1:** Comparison table for CI and CS values of raw and KMnO_4_ treated APFs with other natural fibers.

Fiber name	CI (%)	CS (nm)	References
Untreated AP	46.52	1.9	Present study
KMnO_4_ treated AP	64.47	6.75	Present study
Ceiba pentandra	57.94	22.48	^87^
Celosia argentea	52.54	3.46	^88^
Bauhinia purpurea	65.61	2.57	^89^
Acacia nilotica	44.82	3.21	^90^
Prosopis juliflora	46	15	^69^
Sida cordifolia	56.92	18	^91^
Leucaena leucocephala	63.10	2.33	^92^

### FTIR analysis

**Figure 3:**
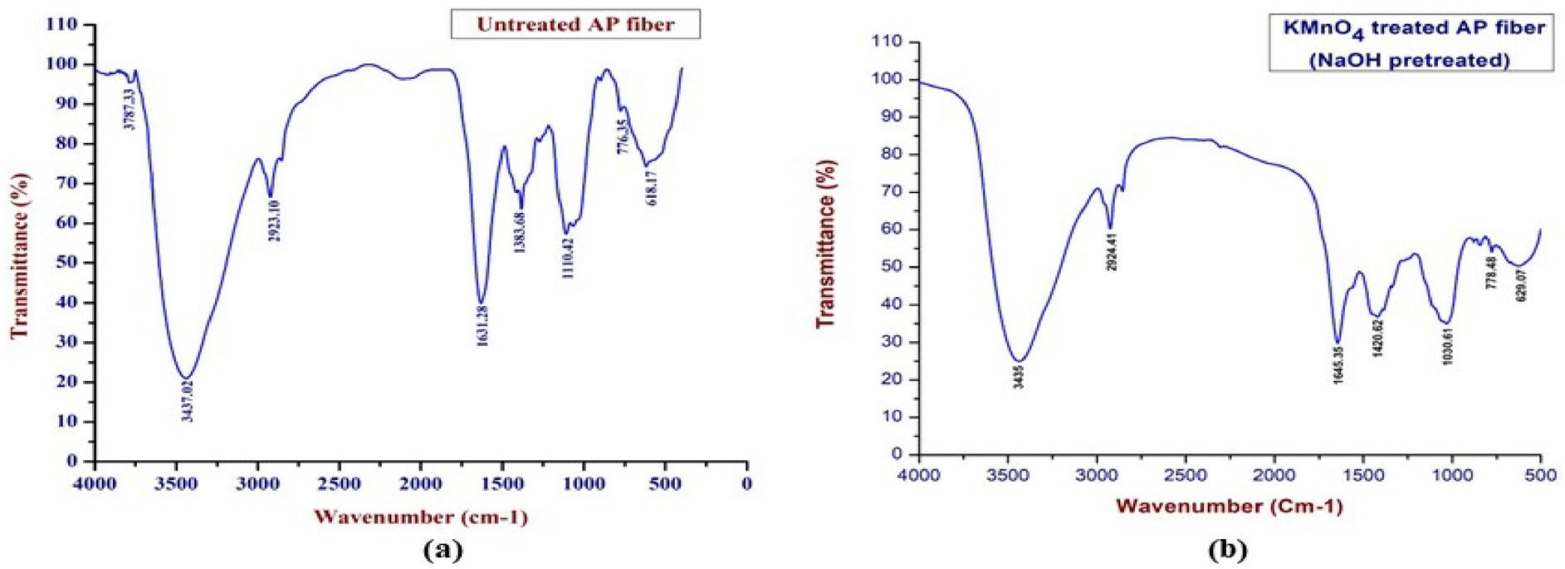
FTIR Spectra of untreated and KMnO_4_ treated AP fiber.

Figure 3 displays the FTIR spectra of the AP fiber that had been treated with KMnO_4_. From graph, the hydrogen-bonded OH stretching group of the water molecules is responsible for the prominent peak at 3435 cm^−1^. The CH stretching vibrations in the cellulose and hemicellulose components are visible in the peak at 2924.41 cm^−1^. The sharp and medium peak, which is associated with the C=H stretching, lies at a height of 1645.35 cm^−1^^93^. A hydrogen bond may be seen at the peak at 1420.62 cm^−1^. The C = O stretching of lignin is what causes the absorption band at 1030.61 cm^−1^ to exist. The presence of saline content may be seen in the lower peak at 778.48 cm^−1^. The peak at 629.07 cm^−1^ demonstrates the region of –OH bending was found at out of plane, proving that the chemical analysis's findings are supported by the elimination of lignin and hemicelluloses from the KMnO_4_ treated APFs. The FTIR vibrational band assignments of untreated and KMnO_4_ treated AP fibers were shown in Table 2.

**Table 2:** FTIR Vibrational band assignments of untreated KMnO_4_ treated AP fibers.

Wave number (cm^−1^)		Vibrational band assignments
KMnO_4_ treated APF	Untreated APF	
–	3787.33	–OH Stretching in Hydrogen bond^93^
3435	3437.02	–Hydrogen bonded of OH stretching in cellulose and/or hemicelluloses^3, 94–97^
2924.41	2923.10	–CH Stretching of Cellulose^97–99^
1645.35	–	–C=O Stretching of α keton^100^
–	1631.28	–CC stretching of lignin^93^
1420. 62	–	–Existence of CH bond^94, 96, 101^
–	1383.68	–Stretching in CH bond^97, 101^
–	1110.42	–COC pyranose ring skeletal vibration of cellulose^102^
1030.61	–	–C=O stretching of lignin^95, 103^
778.48	776.35	Presence of saline content^58, 82^
629.07	618.17	Out of plane of –OH bonding^58, 82^

### SEM analysis

**Figure 4:**
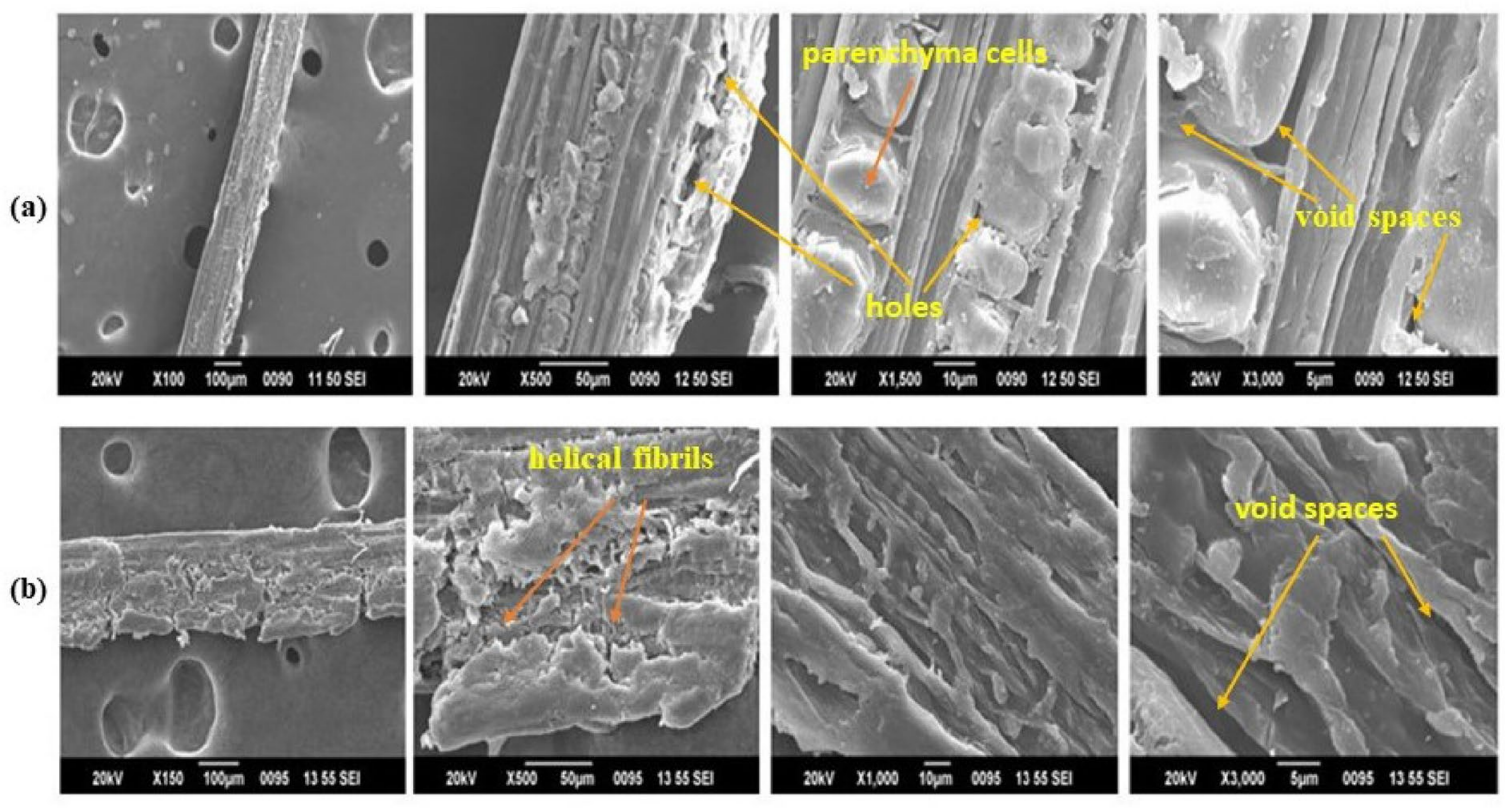
(**a**,**b**) Surface morphology of untreated and KMnO_4_ treated AP fiber under 100, 500, 1500 and 3000 magnification fields.

A test method called scanning electron microscopy uses an electron beam to magnify and examine a material. The surface shape of the untreated and KMnO_4_ treated AP fiber is shown in Fig. 4a,b. It was observed that the APF treated with KMnO_4_ had a rough and disorganised surface shape compared with untreated APFs. It reveals that the hemicellulose content (white layer on the surface of untreated SEM image), void spaces, holes, parenchyma cells and few impurities are visible on its surface. To enhance the interfacial adhesion with polymer matrices, these hemicelluloses and lignin, wax like impurities, amorphous content should be removed, and it was done by KMnO_4_ treatment. Structural properties and its modifications done by this treatment reveals that the experimental fibers can act as a good reinforcement material in composite manufacturing.

### Thermo-gravimetric analysis

**Figure 5:**
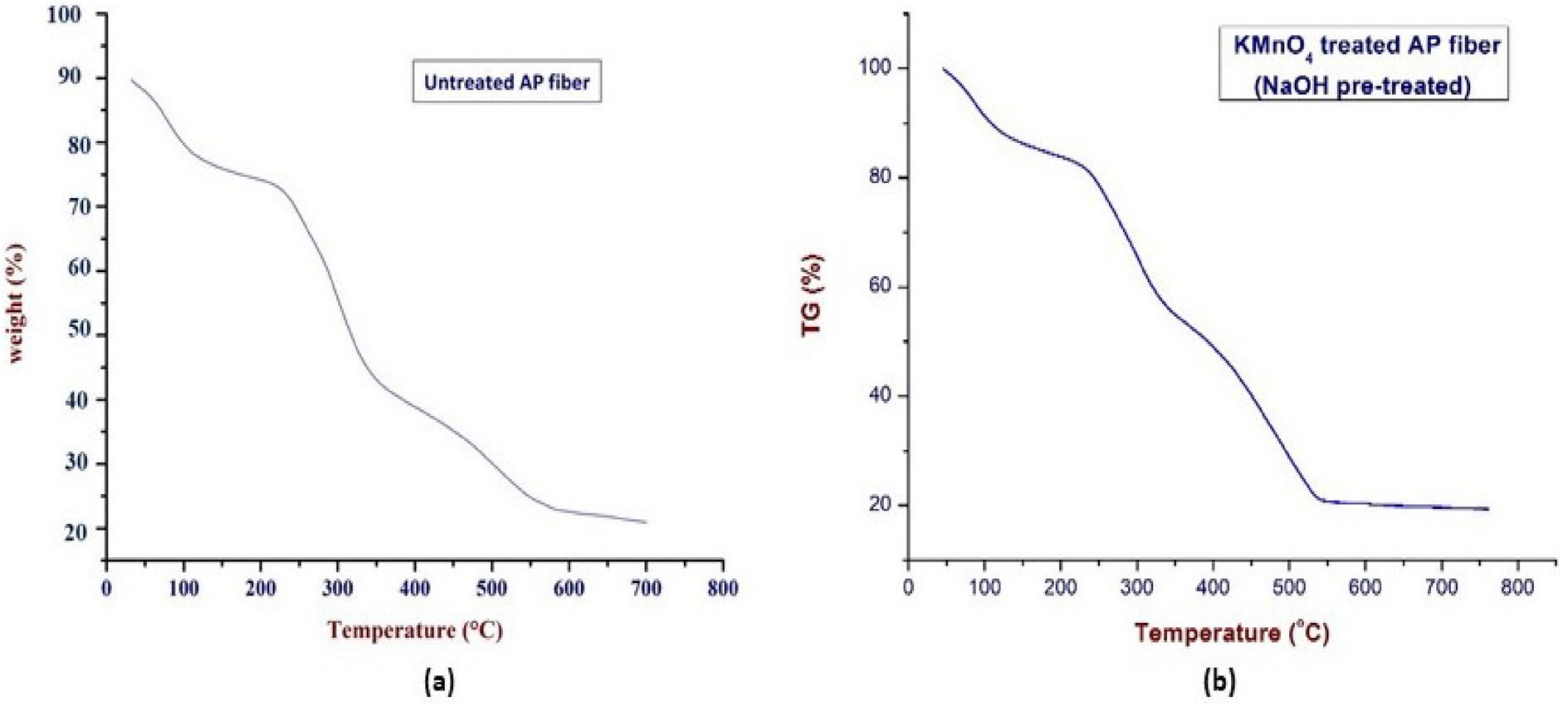
TG curves of untreated and KMnO_4_ treated AP fiber.

**Figure 6:**
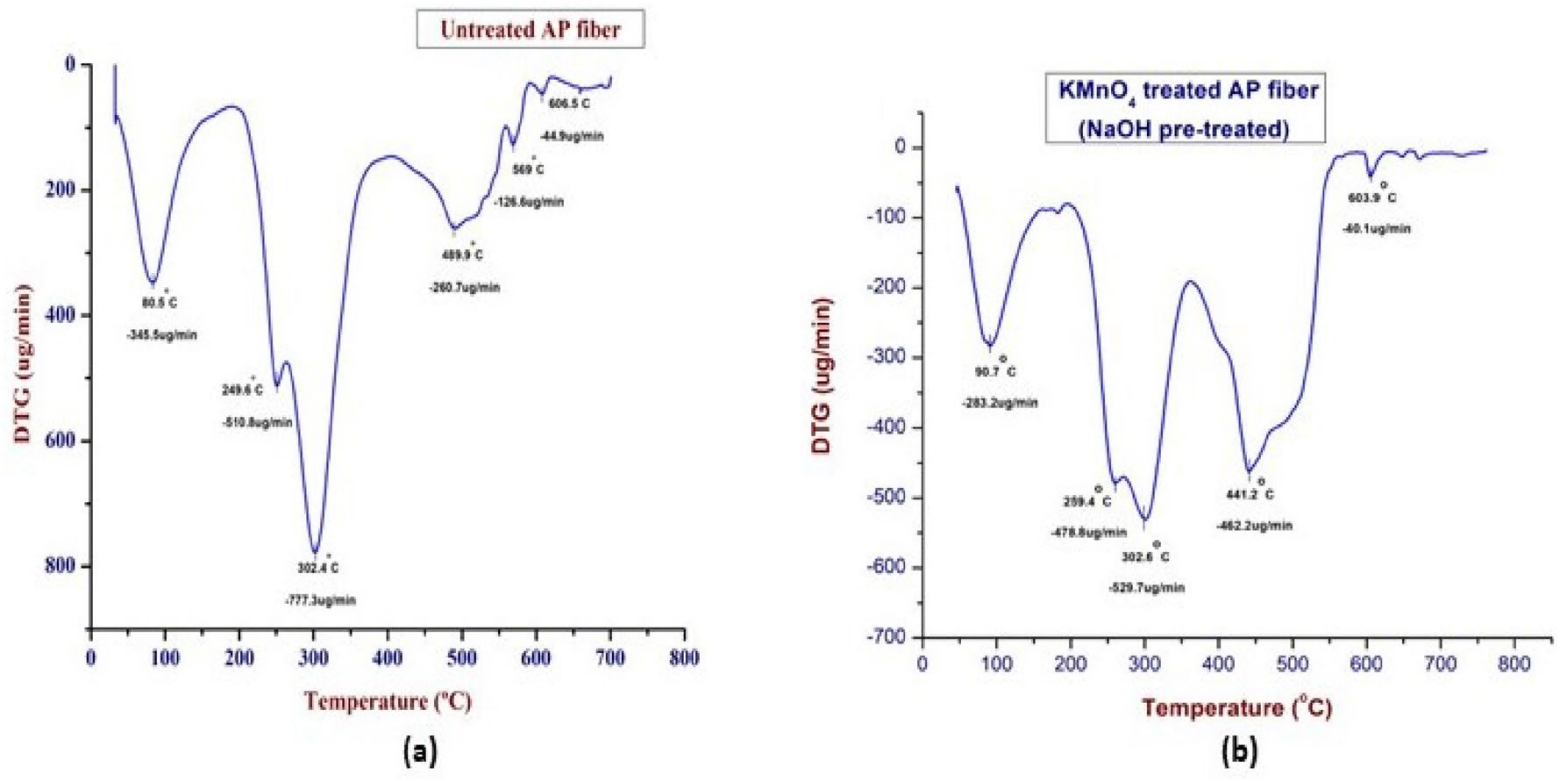
DTG curves of untreated and KMnO_4_ treated AP fibers.

The weight loss of composites in relation to temperature increase was quantified using the thermo-gravimetric analysis. Greater thermal stability results from higher decomposition temperatures^104^. The TG and DTG curves of the KMnO_4_ treated APF sample was shown in Figs. 5 and 6. According to the graph, the degradation peaks are related to moisture evaporation, the breakdown of cellulose, lignin, wax, and other contaminants, as well as hemicellulose. At a temperature of about 40 °C to 120 °C, nearly 12% of weight loss has been occurred, which was mainly depending upon the moisture content in the untreated APF sample. Then the second major degradation occurs between 120 °C and 280 °C temperature range with the reduction of 18.5%. This weight loss has been observed due to the degradation of hemicellulose component. Within the temperature range between 280 °C and 400 °C, 25.46% of weight has been reduced. The destruction of the cellulose content's glycosidic linkages was the cause of the significant weight loss. Another drop was observed at the temperature within the range from 400 °C to 500 °C, nearly 20.93% of loss has occurred, which may be due to the degradation of lignin contents. Finally, degradation between 500 °C and 600 °C, due to the weight reduction of 9.5% due to the loss of wax like substances behind leaving the residue.

In the DTG (Derivative thermo-gravimetric) curve, where the weight loss abruptly occurs at 90.7 °C with a weight loss of 0.283 mg/min, clearly shows the removal of moisture content within the fiber and the second and third degradation peaks were observed at the temperatures of 259.4 °C, 302.6 °C, 441.2 °C with the weight losses of 0.478 mg/min and 0.529 mg/min, 0.462 mg/min. Compared to hemicellulose and cellulose degradation, the thermal decomposition of lignin occurs over a wider temperature range, starts earlier, and goes up to higher temperatures 400 °C^105^. An abrupt drop in the DTG curve, which is related to the thermal breakdown of hemicelluloses and glycosidic linkages in cellulose, serves as an indicator of this^106^. Hemicellulose is a type of polysaccharide that is linked to cellulose and contains various sugar units. Compared to cellulose, it has a higher degree of chain branching but significantly lower levels of polymerization. Hemicellulose thermal degradation occurs before that of cellulose, although its impact is proportionally reduced by the amount of hemicellulose in the fiber^107^.

The next deterioration peak occurred at 603.9 °C with weight losses of 0.040 mg/min, which may have been caused by the breakdown of the fiber's lignin and wax components. Lignin is a complex hydrocarbon polymer that contains both aliphatic and aromatic components^108^. Compared to the thermal decomposition of hemicellulose and cellulose, the thermal decomposition of lignin occurs over a wider range, starts earlier, and goes to higher temperatures. The lesser amount of fiber present, however, also limits its impact^107^. When the temperature ranges from ambient temperature to more than 600 °C, the complex aromatic ring component of the lignin structure decomposes with a minimum weight loss rate^9, 109^. Tables 3 and 4 depicts the comparison between the thermal study (3) and Mass loss at Tmax (4) of untreated and KMnO_4_ treated AP fibers.

**Table 3:** Thermal study of untreated and KMnO_4_ treated AP fibers.

Type of fiber	Temperature during loss (°C)	Weight loss (%)	Thermal stability (°C)	Residual char at 750 °C
Untreated AP fibers	40–120	14.27	328.95	0.072
	120–280	20.41		
	280–400	30.32		
	400–500	11.77		
	500–600	9.91		
KMnO_4_ treated AP fibers	40–120	12	337.47	0.02
	120–280	18.5		
	280–400	25.46		
	400–500	20.93		
	500–600	9.5

**Table 4:** Mass loss at Tmax of untreated and KMnO_4_ treated AP fibres.

Type of fiber	Total mass lost (%)					Tmax (°C)
	1st stage	2nd stage	3rd stage	4th stage	5th stage	
Untreated AP fibers	14.27	34.68	65	76.77	86.88	226.3
KMnO_4_ treated AP fibers	12	30.5	55.96	76.89	86.39	223.5

### Physical analysis

The density of the fiber treated with untreated and KMnO4 was estimated to be 1090 kg/m^3^ and 520 kg/m^3^ (Table 6). Cavities and holes were removed during alkalization^110^. So that, density of the optimally treated APF were slightly decreased. However, the density is slightly lower than that of the Acacia leucophloea 1385 kg/m^3^, coir fiber 1200 kg/m^3^^22^. Due of the uneven profiles of bark fibers, diameter determination in the stem of AP fiber is rather difficult. The measured diameter of the ACF was 299.39 μm which was confirmed from SEM images. The Comparison of diameter and density values of untreated and KMnO_4_ treated APFs with other natural fibers are represented in Table 5.

**Table 5:** Comparison table for diameter and density values of raw and KMnO_4_ treated APFs with other natural fibers.

Fiber name	Density (g/cm^3^)	Diameter (μm)	References
Untreated APF	1.09	299.39	Present study
KMnO_4_ treated APF	1.05	306.72	Present study
Date	0.99	155–250	^57^
Bamboo	0.91	240–330	^111^
Agave	1.20	126–344	^112^
Sea grass	1.50	5	^113^
Palm	1.03	400–490	^57^
Kenaf bast	1.31	65–71	^114^
Curaua	1.40	170	^111, 115^

### CHNS analysis

The Dumas's method^58^, which entails the total and rapid oxidation of the sample by "flash combustion," is the basis for the CHNS analyser, which is used to determine the percentages of carbon, hydrogen, nitrogen, and sulphur in organic compounds. The Dumas method is a technique for calculating the quantity of chemical compounds (elements). This approach is most useful for figuring out how much C, H, N, and S are present in organic compounds, which typically ignite at 1800 °C. The weight percentage of each chemical in the KMnO_4_ treated sample is displayed in Table 6.

**Table 6:** Weight percentage of C, H, N, S in untreated and KMnO_4_ treated AP fiber.

Fiber name	N%	C%	S%	H%
Untreated AP fiber	0.76	43.38	0.51	6.75
KMnO_4_ treated AP fiber	0.80	33.22	ND	4.61

*ND* not detected.

According to CHNS study, untreated AP fibres have a carbon content of 43.38%, however after being treated with permanganate, the carbon content decreases to 33.22%. One of the most vital aspects to alter the mechanical and tribological qualities of the final product is high carbon content in the natural fibers. Both samples can be employed as conductive fillers in dielectric loss materials because of the carbon content is above 30% in both. The average composition of carbon and hydrogen in chicken feather fibres are 47.4% and 7.2%^116^. It is found that the carbon content of coconut shell fibres and sugarcane bagasse fibres are 46.7% and 44.7%. These values are comparable to AP fibers.

### Chemical analysis

Table 7 provides the comparison table of chemical composition of untreated, KMnO_4_ treated with different existing fibers. After pre-treatment, the cellulose content of the plant fibers generally increased. Due to the crystalline areas' altered lattice structures, the APF has a cellulose content of 55.4%^117^. These fibers have lower cellulose levels than Acacia Concinna fiber (59.43 wt%), Acacia leucophloea (68.09 wt% to 76.69 wt%), and Prosopis Juliflora fiber (61.65 wt% to 72.27 wt%)^93^ and larger than that of coir fiber (32–43%) and Ficus leaf fiber (38.1%)^93, 118^. Hemi-Cellulose of the APF was decreased (13.30%) in this treatment. This hemicellulose content was much larger than that of Acacia concinna fiber (12.78%) and Acacia planifrons (9.41%) etc. The diffusion of lignin in KMnO_4_ solution was blamed for the significant change in the lignin concentration (17.75%). However, it is soluble in hot alkali, rapidly oxidised, and condensable with phenol. Lignin is not hydrolyzed by acids^119^. After this treatment, pectin levels similarly dropped (1.9%). Wax content (0.79%) of the KMnO4-treated APF dropped as well, which is a favourable change. As opposed to plant fiber with higher wax content, fiber from plants with reduced wax content can produce excellent interfacial bonds with polymers^120^. The amount of moisture (13.4%) in the KMnO4-treated APF decreased as well. The ash content of the APF, on the other hand, was subtly increased from 10%, which supported the growth of the crystalline component in the fiber^110^.

**Table 7:** Comparison table of chemical compositions of APFs with other existing fibers.

Fiber name	Cellulose (%)	Hemi-cellulose (%)	Lignin (%)	Pectin (%)	Wax (%)	Moisture (%)	Ash (%)	References
Untreated APF	45.68	41.13	24.49	12.19	13.91	0.36	3.13	Present work
KMnO_4_ treated APF	55.4	13.3	17.75	1.9	13.4	0.79	10	Present work
Kenaf fiber	45–57	8–13	21.5	0.6	0.8	6.2–12	2–5	^121, 122^
Wheat husk	36	18	16	–				^123^
Sisal fiber	78	10	8	–	2	11	1	^124^
Agave fiber	68.42	4.85	4.85	–	0.26	7.69	–	^125^
Corn straw fiber	44.5	19.7	25.5	1.4	–	–	–	^121^

### Tensile strength

One of the most often investigated features of natural fiber reinforced composites is tensile strength. When choosing a particular natural fiber for a given application, the fiber strength can be a crucial consideration^126^. Tensile testing of individual technical fibers is a standard method for determining the tensile characteristics of natural fibers^127^. The test was conducted at an ambient temperature of 21 °C, a relative humidity of approximately 65%, and a specimen gauge length of 50 mm^100^. The comparison of tensile strength, young's modulus, micro-fibrillar angle, and breaking elongation of the untreated and KMnO_4_-treated APFs with other natural fibers are shown in Table 8. According to the computed data, the tensile strength of the untreated and KMnO_4_ treated APFs were found to 181.69 MPa and 685 MPa with 6.2%, 4.1% elongation and young's modulus 29.3GPa, 16.707 GPa respectively. The tensile strength of the jute fiber is (400–800 MPa) and its young’s modulus is (10–30 GPa)^128^. As a result, the AP fiber's tensile strength and young's modulus were almost on par with those of jute fiber. The cell walls structure and chemical makeup of bark fibers, particularly the amount of cellulose, have a significant impact on their mechanical properties^129^.

**Table 8:** Comparison table for Tensile strength of untreated and KMnO_4_ treated APFs with other natural fibers.

Fiber name	Tensile strength (MPa)	Young’s modulus (GPa)	Micro-fibrillar angle (°)	Elongation at break (%)	References
Untreated APF	181.69 ± 40	29.30 ± 3	19.67 ± 3	6.2	Present study
KMnO_4_ treated APF	685 ± 60	16.707 ± 7	16.134 ± 5	4.1	Present study
Zea mays	13.243	0.529	12.68	2.5	^59^
Ficus racemosa	270	67.45	–	2.57	^130^
Hemp	690	70	6.2	1.6	^131^
Butea parviflora	198.12	4.4	16.88 ± 9.87	4.5	^132^
Heteropogon contortus	476 ± 11.6	48 ± 2.8	14.53 ± 0.53	–	^133^
Cissus quadrangularis	200.39	4.89	–	3.57–8.37	^51^
Banana	700–800	27–32	–	–	^133^

## Conclusion

The paper discusses the outcomes of analyses performed on the KMnO_4_-treated AP fiber using X-ray diffraction (XRD), Fourier Transform Infrared (FTIR), scanning electron microscopy (SEM), thermo-gravimetric analysis (TGA), and mechanical (tensile strength) analysis.

1. The mechanical analysis of AP fiber makes it a dependable and long-lasting material for building composite fiber materials and fiber reinforced concrete for use in construction. Due to the orientation of the fibers, AP fibers have superior mechanical properties (such strength and young's modulus), which improves their capacity to handle loads and stress.
2. The KMnO_4_-treated AP fiber's X-ray diffraction (XRD) patterns revealed a semi-crystalline structure, with a crystallinity index of 51.63%. These findings imply that the KMnO_4_ treatment was successful in eliminating impurities and raising the AP fiber's crystallinity index. The crystallite size of the KMnO_4_-treated AP fiber was 1.05 nm. Overall, the results of this study provide valuable information on the effects of KMnO_4_ treatment on the structure, stability and mechanical analysis of AP fiber.
3. FTIR spectral analysis revealed the existence of lignin, saline content, and components of cellulose and hemicellulose. Scanning electron microscopy revealed that the KMnO_4_-treated AP fiber's surface was rough and disordered, with amorphous content and impurities clearly apparent. Chemical analysis outcomes showed that APF has the higher cellulose (55.4%) and lesser hemicellulose (13.3%) content.
4. The degradation of moisture, cellulose, lignin, wax, and other impurities, as well as hemicellulose, corresponded to the peaks in the thermo-gravimetric study. This thermo-gravimetric analysis showed that the KMnO_4_ treated AP fiber has a higher thermal stability (337.47 °C), with higher decomposition temperatures (223.5 °C), making it a potential candidate for use in composite materials.
5. This fiber's overall structure and stability have improved as a result of the KMnO_4_ treatment. Therefore, with further purification, AP fibre has the potential to be used as a reinforcement material in the creation of composites. All the above findings and lower density (520 kg/m^3^) of the APF would make them suitable for lightweight composite materials.

The experiments conducted on KMnO4 treated Acacia pennata fibers show remarkable mechanical properties, such as tensile strength of 685 MPa and a Young's modulus of 16.707 GPa. The high tensile strength of Acacia pennata fibers has a possibility of utilizing as particle replacement of cementitious material in the construction as well as interior structural components. In the current practices many natural fibers are utilized in the construction sector. Furthermore, these composites have high potential to be applied in the sandwish structure due to its light weight with higher flexural properties. The fibers microfibrillar angle of 16.134º and break elongation of 4.1% further support their suitability for these applications. However, it is crucial to evaluate these results against industry standards to fully assess the potential of the Acacia pennata fibers applications. Further research should be conducted to examine their feasibility and performance against existing materials in the construction and composite industries.

## Data Availability

The datasets generated during and/or analysed during the current study are available from the author and corresponding author on reasonable request.
